# Subjective Stress, Salivary Cortisol, and Electrophysiological Responses to Psychological Stress

**DOI:** 10.3389/fpsyg.2016.00229

**Published:** 2016-02-18

**Authors:** Mingming Qi, Heming Gao, Lili Guan, Guangyuan Liu, Juan Yang

**Affiliations:** ^1^Faculty of Psychology, Southwest UniversityChongqing, China; ^2^School of Psychology, Liaoning Normal UniversityDalian, China; ^3^College of Electronic and Information Engineering, Southwest UniversityChongqing, China

**Keywords:** psychological stress, mental arithmetic task, dissociable effect, N1, P2

## Abstract

The present study aimed to investigate the subjective stress, salivary cortisol, and electrophysiological responses to psychological stress induced by a modified version of a mental arithmetic task. Fifteen participants were asked to estimate whether the multiplication product of two-decimal numbers was above 10 or not either with a time limit (the stress condition) or without a time limit (the control condition). The results showed that participants reported higher levels of stress, anxiety, and negative affect in the stress condition than they did in the control condition. Moreover, the salivary cortisol level continued to increase after the stress condition but exhibited a sharp decrease after the control condition. In addition, the electrophysiological data showed that the amplitude of the frontal-central N1 component was larger for the stress condition than it was for the control condition, while the amplitude of the frontal-central P2 component was larger for the control condition than it was for the stress condition. Our study suggests that the psychological stress characteristics of time pressure and social-evaluative threat caused dissociable effects on perception and on the subsequent attentional resource allocation of visual information.

## Introduction

Psychological stress is defined as a state of perceived threat to homeostasis (Pacak and Palkovits, 2001). The neuroendocrine response to psychological stress is primarily transduced through the hypothalamus–pituitary–adrenal (HPA) axis and sympathetic adrenal medulla axis (Foley and Kirschbaum, 2010). Recently, we investigated the time course of psychological stress by instructing participants to complete both an easy mental arithmetic task (control condition) and a difficult mental arithmetic task (stress condition) under time pressure while their electrophysiological data were recorded (Yang et al., 2012). The results of our previous study showed that the amplitude of the occipital N1 component was more negative for the control relative to stress condition, which might reflect the process of discriminating between big and small numbers in the control task, and the latency of the frontal P2 component was shorter for the stress relative to control condition, which might reflect the faster orienting and processing of visual information in the stress task (Yang et al., 2012). Similarly, some studies have found that stress alters early sensory processing, as reflected by increased N1 amplitudes (Shackman et al., 2011; Elling et al., 2012; Löw et al., 2015). These findings suggest that psychological stress may primarily modulate cognitive processing at a relatively early stage. However, our previous study (Yang et al., 2012) had several limitations that were related to the experimental design. First, and the most importantly, the differential neural activity that was observed across conditions might have been modulated not only by the psychological stress but also by the task difficulty, which differed between the two conditions. Specifically, participants were asked to estimate whether the multiplication product of two two-decimal numbers was above 10 or not. In the control condition, the multiplied numbers resulted in products that were significantly greater than or less than 10 (e.g., 1.23 × 0.54 = 0.66). However, the multiplied numbers in the stress condition had products that were very close to 10 (e.g., 2.15 × 4.92 = 10.58). Consequently, more mental effort (load) was required to complete the more difficult task in the stress condition relative to the easier task in the control condition. Second, because the uncontrollability caused by performing the arithmetic task under time pressure is the characteristic and important part of classical psychological stress paradigms (Kirschbaum et al., 1993; Dedovic et al., 2005; Wang et al., 2005, 2007; Ying et al., 2011), the time pressure for the control condition would still be perceived as stressful. Third, the control task and the stress task were performed successively, therefore, participants’ performances in the stress condition might have been affected by the elevated fatigue level and the emotional state evoked by the control condition.

The present study was designed to avoid these limitations. Firstly, the task difficulty was counter-balanced across conditions. Specifically, the same set of multiplication formulas was adopted for the control and stress conditions, which eliminated the differences in the stimuli and mental load between the two conditions. Secondly, we manipulated the duration of multiplication formula separately in order to differentiate the stress level in the two conditions, with the stress condition having shorter durations and the control condition having longer durations. Thirdly, we inserted a rest block (baseline2, 10 min) between the stress and control blocks to alleviate the effects of psychological stress on the subsequent control condition. Moreover, we added feedback that showed the participants’ individual reaction time (RT) and the average RT during the stress block, which served as a social-evaluative threat (Dedovic et al., 2005, 2009; Pruessner et al., 2008). Using this design, we were able to establish a stressful situation characterized by uncontrollability and social-evaluative threat.

Therefore, using the new design, the present study aimed to further investigate the neural mechanism of the effects of psychological stress. Event-related potentials (ERPs) were recorded while participants performed a mental arithmetic task. Stress can fundamentally alter neural responses to incoming information, and modulates early sensory information processing (Shackman et al., 2011). Some studies have suggested that acute stress may elicit a state of heightened vigilance and arousal (Wang et al., 2005) and may sensitize early sensory encoding (Shackman et al., 2011; Löw et al., 2015). Therefore, we predicted that the level of vigilance and arousal would be higher for the stress condition than for the control condition. The N1 component reflects sensory perception and is sensitive to the level of vigilance, and more negative amplitudes would be evoked as the vigilance level increases (Naatanen and Picton, 1987; Ohno et al., 2000; Sponheim et al., 2006; Shackman et al., 2011). Accordingly, an enhanced N1 component would be expected for the stress versus control condition.

In addition, some studies have suggested that psychological stress shifts the balance of attention away from a task-directed mode to a sensory-vigilance mode (Bishop, 2007; Arnsten, 2009; Shackman et al., 2011). Specifically, selective attention processes are modulated by stress (Shackman et al., 2011; Sänger et al., 2014; Löw et al., 2015). It is also possible that the time pressure might narrow the focus of attention and affect the efficiency of perceptual processing (Kowalski-Trakofler et al., 2003; Dambacher and Hübner, 2015). Therefore, we predicted that attentional processing would be negatively affected in the stress versus control condition. Accordingly, since the P2 component is related to attentional allocation (Thorpe et al., 1996; Yuan et al., 2011), with increasing attention resulting in increased P2 amplitudes (Smid et al., 1999; González-Garrido et al., 2015), a reduced P2 component was expected for the stress versus control condition due to the effect of psychological stress.

## Materials and Methods

### Subjects

Fifteen volunteer participants were recruited from the local university (eight females, mean age = 21.3 years, *SD* = 1.4) and were pre-screened with the Beck Depression Inventory (Beck, 1967). All of the female participants were in the follicular phase (Kirschbaum et al., 1999; Jayasinghe et al., 2015). All participants were right-handed, and had normal or corrected-to-normal vision. All participants signed an informed consent form and were paid for their participation. This study was approved by the Research Ethics Committee of the Southwest University of China and was performed in accordance with the ethical guidelines of the Declaration of Helsinki.

### Materials

A set of multiplication formulas containing 350 arithmetic expressions (e.g., 4.94 × 2.01) was adopted in the current study. Data from our pilot study (15 subjects, nine females; mean age = 22.7 years, *SD* = 0.9) revealed that the participants’ RTs were longer for the control condition than they were for the stress condition [*t*(14) = 5.53, *p* < 0.001], while the accuracy was higher for the control condition than it was for the stress condition [*t*(14) = 6.35, *p* < 0.001].

### Procedure

**FIGURE 1:**
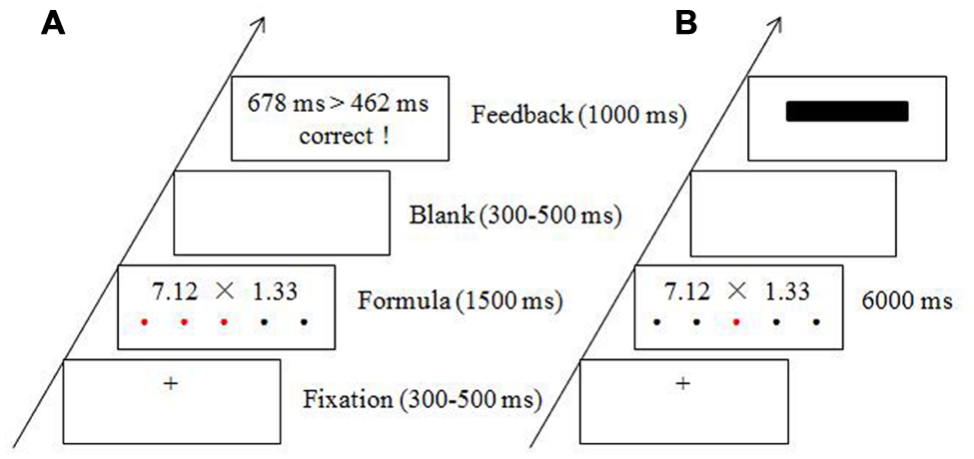
**Experimental procedure and sample materials for the stress condition (A) and the control condition (B)**.

The protocol in the current study consisted of four experimental blocks during which electroencephalography (EEG) data were collected. Each block lasted for 10 min. The first and third blocks were the baseline blocks, in which participants were asked to rest, without performing any tasks. The second and fourth blocks were the stress and control conditions, respectively, which were conducted in a fixed order. In order to collect participants’ continuous salivary cortisol samples, three rest blocks were placed at the end of the experiment, and EEG data were not recorded during these blocks. For the modified mental arithmetic task, participants were asked to estimate whether multiplying two two-decimal numbers would result in a product that was above 10 or not within a time limit. Prior to beginning the experiment, all participants were instructed to respond as quickly and accurately as possible by pressing the corresponding response key. In the stress condition, participants were given 1500 ms to complete the mental arithmetic task. The time limit was enforced for each trial and the elapsed time was indicated by red dots that progressed from left to right on the computer screen. As soon as a response was submitted, the formula disappeared and a blank screen was presented. Then, meaningful feedback was presented, which consisted of a comparison between the participant’s individual RT and the average RT and the correctness of the response, with the RT comparison on top and the correctness on the bottom (see **Figure 1A**). In the control condition, participants were given 6000 ms to complete the same mental arithmetic task. To ensure consistency between the stress and control conditions regarding the formatting of the stimuli, red dots were also presented below the multiplication formula in the control condition but in a pseudo-random order. The formula disappeared once a response was submitted. The meaningful feedback was replaced by a black rectangle (see **Figure 1B**). In addition, participants were told that their movements and performances were being monitored throughout the stress block, while no monitoring or comparisons were being performed during the control block.

In each trial, a random fixation cross (+, 300–500 ms) appeared at the center of the screen. Then, the mental arithmetic task was presented for up to 1500 ms in the stress condition and up to 6000 ms in the control condition. After a blank screen was presented for 300–500 ms (blank timing was random), the feedback (or black rectangle in the control) was displayed for 1000 ms (see **Figure 1**).

During the experiment, participants sat comfortably, approximately 80 cm from a computer screen, in an electrically shielded room. All stimuli were presented on a white background and were displayed at the center of a 17-inch screen using E-Prime 1.0 (Psychology Software Tools, Inc., Sharpsburg, USA). Subjects were instructed to try and minimize blinking, to keep movement to a minimum, and to fix their eyes on the center of the screen and avoid looking down at their fingers when responding.

Self-reports of the participants’ stress levels (on a scale of 1–5) were obtained and saliva samples were collected (using a Salivette sampling device; Sarstedt, Rommelsdorf, Germany) immediately after the scalp cap was in place and after each block. Thus, data were collected at eight time points in total. Saliva samples were stored at –80°C until the assays were performed. Furthermore, participants’ anxiety levels and emotional states were assessed by the State Trait Anxiety Inventory (Wang et al., 1999) and Positive and Negative Affect Scale (Watson et al., 1988), respectively, immediately after the stress and control blocks. All experiments were performed between 14:00 and 16:00 to control for the diurnal fluctuations in cortisol levels (Chen et al., 2014).

### Behavioral and Physiological Data Analysis

The salivary cortisol levels were assayed using an enzyme immunoassay kit. Paired-sample *t*-tests were performed on the state anxiety, emotional state, RT, and accuracy data. Self-reported stress data and salivary cortisol were analyzed with repeated measures ANOVAs using SPSS 16.0 (SPSS, Chicago, IL, USA).

### Electrophysiological Recording and Analysis

Brain electrical activity was recorded at 64 scalp sites using tin electrodes mounted in an elastic cap (Brain Products, Germany). It was placed on the scalp according to the 10–20 system positions with the reference on the left and right mastoids. Vertical and horizontal electrooculograms (EOG) were recorded from above and below the right eye and at the right and left outer canthi, respectively. The inter-electrode impedance was maintained below 5 kΩ at all times. The electroencephalogram (EEG) and EOG were amplified using a 0.05–100 Hz bandpass, and continuous sampling was conducted at 500 Hz/channel during on-line recording.

Raw EEG data were processed oﬄine using BrainVision Analyzer version 1.05 (Brain Product GmbH; Gilching, Germany). For the data analysis, ERPs time-locked to the onset of the multiplication formulas were re-referenced algebraically to the average of the left and right mastoids. After ocular correction (Gratton et al., 1983), EEGs were digitally filtered with a 30-Hz low-pass filter with a 24 bit analog-to-digital converter. The behavioral data showed that it took about 500 ms (in the stress condition) for participants to make a response to the formulas. Thus, the EEGs were segmented into 700-ms epochs surrounding the onset of the stimulus, and then baseline-corrected with respect to the 200 ms pre-stimulus. Trials contaminated with electrooculogram artifacts (mean electrooculogram voltage exceeding ±80 μV) or those with artifacts due to amplifier clipping, bursts of electromyographic activity, or peak-to-peak deflection exceeding ±100 μV were excluded from averaging. EEGs recorded in the two conditions were averaged separately for each participant.

Based on the grand averaged ERPs and topographical maps of the difference waveforms (as shown in **Figure 3**, the maximum difference appeared over frontal-central scalps), the following frontal to central scalp electrode sites were selected for analysis in the present study: Fz, F1, FC1, FCz, Cz, C1, F2, FC2, and C2. These sites correspond to those used in previous ERP studies on stress (Bertsch et al., 2011; Yang et al., 2012). Therefore, separate analyses were performed using two-way repeated measures analyses of variance (ANOVAs) for the following variables: peak latency and amplitude of N1 (80–130 ms) and P2 (130–250 ms) at nine electrode sites (Fz, F1, FC1, FCz, Cz, C1, F2, FC2, and C2). For the N1 epoch, peak detection was performed semi-automatically using the Brain Vision Analyzer software (Brain Product GmbH). The N1 peak was defined *a priori* as the most negative value between 80 and 130 ms, and the P2 peak was defined as the most positive value between 130 and 250 ms. This corresponds to the typical latency range of the N1 (Escera et al., 2000; Reinvang et al., 2000) and P2 (Sponheim et al., 2006; O’Toole and Dennis, 2012) components. The peak latencies were defined as the time points at which the ERP components reached their maximum amplitude within the given time range. A complementary analysis was also performed on the peak-to-peak P2-N1 amplitudes by subtracting the peak value of the N1 component from the P2 peak. All effects with more than one degree of freedom were adjusted for sphericity violations using the Greenhouse–Geisser correction. Main effects were followed by Bonferroni-corrected pairwise comparisons.

## Results

### Behavioral and Physiological Data

**FIGURE 2:**
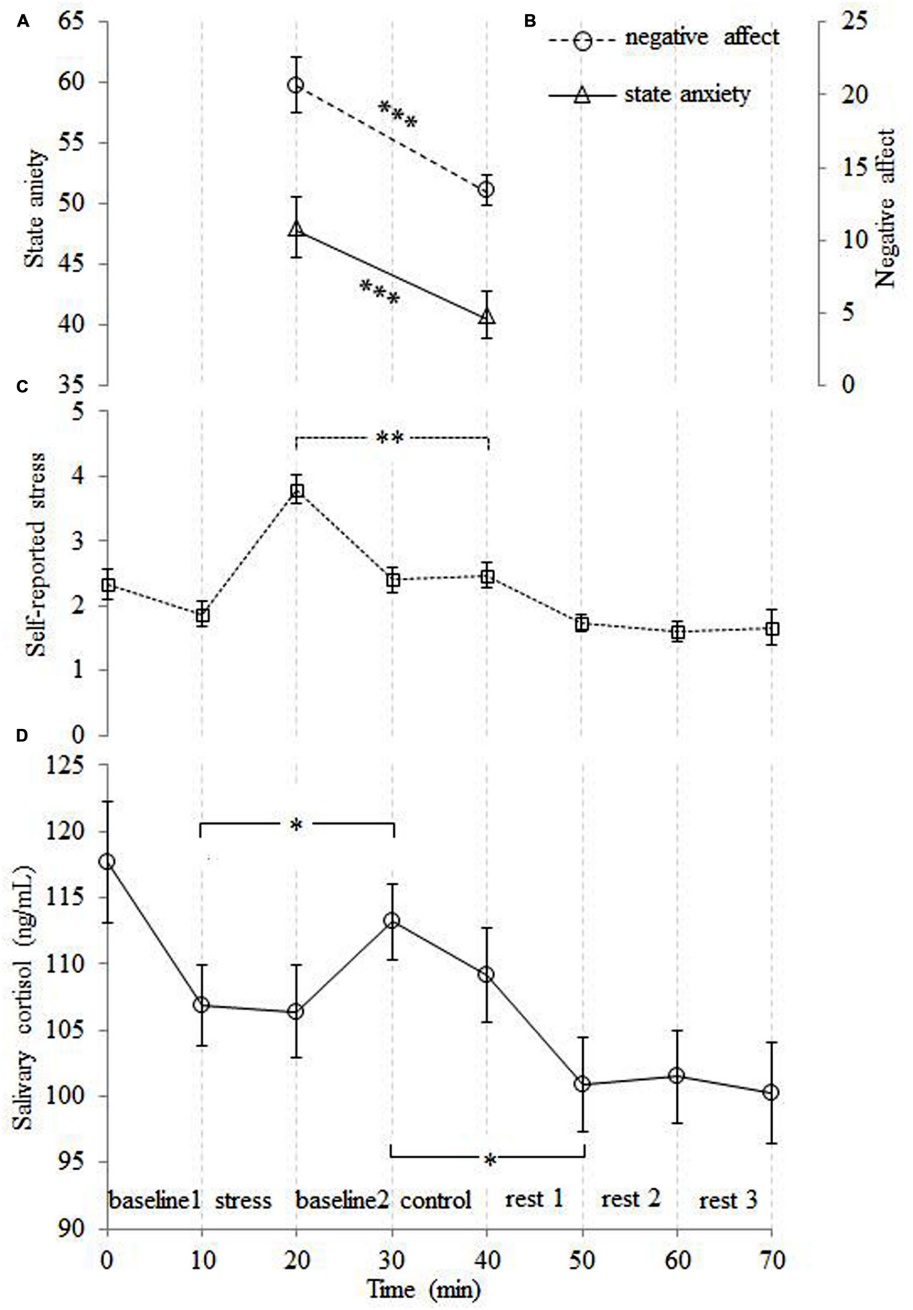
**The time courses of the averaged subjective ratings of state anxiety (A), negative affect (B), self-reported stress (C) and salivary cortisol levels (D)**. The level of subjective stress was higher for the stress than control condition. The level of cortisol was obviously increased after the stress condition, and then decreased sharply after the control condition. Note that the peak in the salivary cortisol level lags behind the other measures. Error bars represent the standard error of the mean. ^∗^*p* < 0.05, ^∗∗^*p* < 0.01, ^∗∗∗^*p* < 0.001.

Both the level of state anxiety [*t*(14) = 4.22, *p* = 0.001] and the level of negative affect [*t*(14) = 6.31, *p* < 0.001] were higher for the stress relative to the control condition (see **Figures 2A,B**). Self-reported stress and salivary cortisol data were analyzed using repeated-measures ANOVAs, with time point as the within-subjects factor. The self-reported stress level analysis demonstrated a main effect of time point [*F*(7,98) = 19.70, *p* < 0.001]. Pairwise comparisons revealed that the level of self-reported stress after stress cessation was higher than the stress level at all other time points (all *p*s < 0.01; see **Figure 2C**). A main effect of time point was also found for the salivary cortisol [*F*(7,98) = 3.42, *p* = 0.012]. This demonstrated that salivary cortisol reached its peak 10 min after stress cessation (*p* = 0.044), which is consistent with the expected time lag between peripheral cortisol and behavioral measures (Kirschbaum et al., 1993; Wang et al., 2005). The salivary cortisol then exhibited a sharp decrease 10 min after the control condition (*p* < 0.05; see **Figure 2D**).

For the RT data, there was a significant difference between the two conditions [*t*(14) = 15.79, *p* < 0.001], with longer RTs for the control condition (774 ± 157 ms) versus the stress condition (511 ± 50 ms). For the accuracy data, the difference between the conditions was also significant [*t*(14) = 6.39, *p* < 0.001], with the control condition having a higher accuracy (0.81 ± 0.03) than the stress condition (0.68 ± 0.04).

### Electrophysiological Data

**FIGURE 3:**
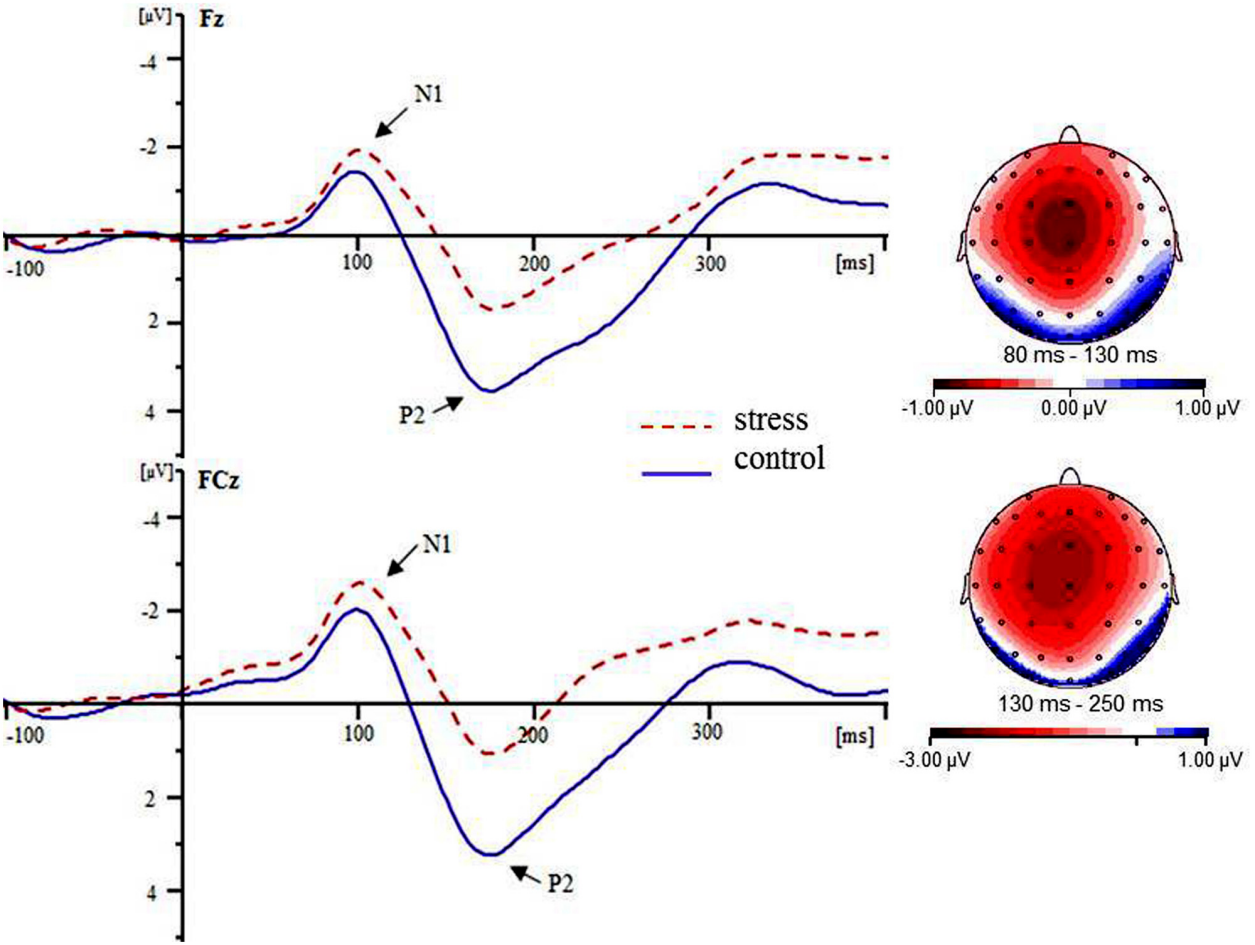
**Grand averaged ERPs for the stress condition (red dotted lines) and the control condition (blue solid lines) at the Fz and FCz electrode sites**. The topographic maps indicate the distribution of the condition type effect during the N1 (80–130 ms) and P2 (130–250 ms) time windows.

The grand averaged maps for the two conditions are shown in **Figure 3**. During the N1 (80–130 ms) time window, for the N1 amplitude, the main effect of condition was significant [*F*(1,14) = 6.77, *p* = 0.021]. The condition × electrode site interaction was also significant [*F*(8,112) = 2.91, *p* = 0.033]. Simple effects analysis revealed that the amplitude of N1 was more negative for the stress versus control condition at all electrode sites [all *F*(1,14) > 5.46, *p*s < 0.05] except F2 [*F*(1,14) = 1.22, *p* = 0.228]. For the latency of N1, neither the main effect of condition nor interaction of condition × electrode site was significant [all *F*s < 2.12, *p*s > 0.12]. During the P2 (130–250 ms) time window, for the P2 amplitude, the main effect of condition was significant [*F*(1,14) = 115.93, *p* < 0.001]. The condition × electrode site interaction was also significant [*F*(8,112) = 3.25, *p* = 0.019]. Simple effects analysis revealed that the amplitude of P2 was more positive for the control versus stress condition at all electrode sites [all *F*(1,14) > 54.93, *p*s < 0.001]. To rule out that the condition effect on P2 was due to variations already present at the level of N1, we ran a peak-to-peak analysis to complement the baseline-to-peak analysis. The main effect of condition was significant when the N1 peak amplitude was subtracted from the P2 peak [*F*(1,14) = 60.29, *p* < 0.001]. The condition × electrode site interaction was not significant (*p* > 0.05). These findings suggest that the difference in the N1 amplitude did not contribute to the P2 amplitude differences. For the latency of P2, neither the main effect of condition nor interaction of condition × electrode site was significant (all *F*s < 1.67, *p*s > 0.20).

## Discussion

The current study aimed to further investigate the subjective stress, salivary cortisol, and electrophysiological responses to psychological stress induced by a modified version of a mental arithmetic task. The stressful situation was characterized by uncontrollability (induced by the time pressure) and social-evaluative threat (evoked by the immediate meaningful feedback). Our results showed that the mental arithmetic task successfully evoked psychological stress, as measured by participants’ subjective stress and endocrine responses.

Compared to the levels in the control condition, higher levels of self-reported stress, state anxiety and negative affect were reported by the participants in the stress condition. This is consistent with previous well-established studies, in which the psychological stress was induced by serial subtraction with verbal feedback (Kirschbaum et al., 1993; Wang et al., 2005, 2007) or computerized mental arithmetic with built-in social evaluation (Dedovic et al., 2005). Higher levels of cortisol were also found in our study following the stress versus control condition. Because the release of cortisol is not instantaneous, the peak in salivary cortisol level lags behind other measures (i.e., self-reported stress). Specifically, cortisol levels gradually increase within a few minutes after stimulation onset and reach peak concentrations 10–30 min after stress cessation (Kirschbaum et al., 1993; Dedovic et al., 2005; Wang et al., 2005, 2007; Foley and Kirschbaum, 2010). In line with this notion, we found that the cortisol level continued to elevate after the stress condition and then decreased sharply after the control condition, reflecting the typical stress-related activity increases in the HPA axis.

The lower performance (i.e., accuracy) for the stress relative to the control condition was probably due to the negative effects of psychological stress on attention processes. Some studies have shown that the focus of attention shrinks under time pressure, participants adopt a simpler mode of information processing in which alternatives are not explored fully and certain important cues are used to determine the decision (Kowalski-Trakofler et al., 2003). In other words, the time pressure stress narrows the focus of attention and affects the efficiency of perceptual processing (Dambacher and Hübner, 2015). Cain et al. (2011) found that anticipatory anxiety reduced the accuracy of target detection, which reflected the narrowing of attention (Easterbrook, 1959). In the present study, the lower accuracy for the stress condition might be due to the reduced allocation of attentional resources to the formulas. Participants might have responded to the formulas based on critical issues and elements (i.e., just the first numbers and not the decimals) under the time pressure. This is consistent with previous studies that showed faster, less precise perceptual processing under stress (Bertsch et al., 2011).

With the merit of time resolution, the ERP results revealed that the N1 amplitude was more negative for the stress versus control condition over the frontal-central scalp. However, our previous study (Yang et al., 2012) demonstrated a more negative occipital N1 component for the control versus stress condition. In our previous study, the stimuli in the control and stress condition were different, therefore, the differential N1 amplitude might have reflected the different discriminatory processes for the big or small numbers between the control and stress conditions due to the different stimuli (Yang et al., 2012). However, the larger N1 for stress than control condition in the present study was not due to the stimuli, because the stimuli were the same for both conditions. Some studies have suggested that the anterior visual N1 component reflects sensory perception and is sensitive to the level of vigilance (Naatanen and Picton, 1987; Ohno et al., 2000; Sponheim et al., 2006; Shackman et al., 2011). Moreover, studies have shown that acute stress elicits a state of heightened vigilance and arousal (Wang et al., 2005; Shackman et al., 2011). In this study, the shorter duration of stimuli together with the immediate meaningful feedback that was presented following the participants’ responses might have created a competitive atmosphere for the stress relative to the control condition. A stronger sense of uncontrollability was produced and a higher level of vigilance was upheld in the stressful situation, which could be considered as a preparation for the timely detection of threat. Therefore, the N1 amplification that was noted in the stress condition might suggest that increased vigilance and augmented sensory intake were elicited by the threatening environments (Davis and Whalen, 2001; Shackman et al., 2011).

A larger frontal-central P2 component was found for the control versus stress condition in the present study. However, no amplitude difference was found during P2 epoch across conditions in our previous study, instead, a shorter P2 latency was found for the stress versus control condition (Yang et al., 2012). In our previous study, the multiplication formulas were more difficult in the stress condition than they were in the control condition, thus the shorter P2 latency related to the faster orienting and processing of visual information might have been due to the differences in task difficulty. In the present study, the differential P2 components were likely not due to differences in task difficulty, because the task difficulty was counter-balanced between the two conditions. Some studies have suggested that stress disrupts the mechanisms involved in attention processing (Plessow et al., 2012; Sänger et al., 2014; Dambacher and Hübner, 2015), and that a larger P2 is always associated with enhanced selective attention (Smid et al., 1999; Bergström et al., 2007; Lenartowicz et al., 2014; Löw et al., 2015). Therefore, the reduced P2 for the stress relative to the control condition might suggest that the stress had a negative effect on the perceptual and attention processing of stimuli. Specifically, due to the time pressure and social-evaluative threat, it is likely that less attentional resources were allocated to the formulas for the stress relative to the control condition. Such an interpretation is supported by our behavioral results, which showed faster RTs and less accurate responses for the stress versus control condition. This is consistent with the study which suggested that decreased frontal-central P2 amplitude was associated with faster but less precise attention processing (Bertsch et al., 2011).

There are several limitations of the current study that should be addressed in a future study. First, both our previous study and the current study aimed to explore the neural substrates associated with the psychological stress generated when the multiplication of two-decimal numbers is processed, which reflects a fast neural response to stimuli. However, psychological stress is defined as a state of perceived threat to homeostasis (Pacak and Palkovits, 2001). Although psychological stress influences the early ERP components (i.e., N1, P2), and these components are sensitive to the perception and attentional allocation processes, it is unclear how these processes were directly tested in the present study. In other words, the stress-related alterations in perception and attention remain to be directly tested in the future. Furthermore, future studies should investigate the neural responses to the psychological stress by recording the brain oscillations during the resting periods after the psychological stress using the EEG, which is one of the most direct and effective physiological measures for assessing the state of arousal. Moreover, the sample size of the present study was not sufficient for performing a correlation analysis of the physiological and electrophysiological data, which is important for making our claims more convincing, thus, further studies should employ a larger sample size in order to conduct correlation analyses. In addition, as the novelty of the mental arithmetic task itself might cause effects on the participants’ stress levels and emotional states, the order of the stress and control blocks should be counter-balanced across participants in future studies.

## Conclusion

Our findings suggest that the psychological stress increased the levels of subjective stress and salivary cortisol, and seemed to have a dissociable effect on the early stages of cognitive processing. Specifically, an enhanced N1 component was found for the stress relative to the control condition. This might suggest that vigilance and sensory intake are augmented under stress. Furthermore, a reduced P2 amplitude was found for the stress versus the control condition, which might suggest that the stress had a negative effect on the allocation of attentional resources.

## Author Contributions

MQ, JY designed the experiment. MQ, LG conducted the experiment and analyzed the data by supervision of JY and GL. MQ, HG wrote the manuscript. All authors edited and revised manuscript and approved final version of manuscript.

## Conflict of Interest Statement

The authors declare that the research was conducted in the absence of any commercial or financial relationships that could be construed as a potential conflict of interest.
